# Production and Characterization of Glutathione-Chitosan Conjugate Films as Systems for Localized Release of Methotrexate

**DOI:** 10.3390/polym11122032

**Published:** 2019-12-07

**Authors:** Yhors Ciro, John Rojas, Cristian J. Yarce, Constain H. Salamanca

**Affiliations:** 1 Department of Pharmacy, School of Pharmaceutical and Food Sciences, University of Antioquia, 67 Street No. 53–108, Medellín 050025, Colombia; jrojasca@gmail.com; 2 Laboratorio de Diseño y Formulación de Productos Químicos y Derivados, Departamento de Ciencias Farmacéuticas, Facultad de Ciencias Naturales, Universidad ICESI, Calle 18 No. 122–135, Cali 760035, Colombia; cjyarce@icesi.edu.co

**Keywords:** casting-evaporation, drug delivery systems, glutathione-chitosan conjugates methotrexate, surface characterization, polymer films

## Abstract

Cancer is one of the most serious public health problems that affect humanity. Diverse delivery systems of anticancer drugs have been developed to enhance the treatment effectiveness and patient compliance. Thus, drug delivery systems from polymeric films could be an interesting and promising alternative, especially for skin chemotherapeutics. In this work, polymeric films based on glutathione-chitosan conjugates with degrees of thiolation of 4.4%, 5.1% and 7.0% were synthetized by casting-evaporation method and subsequent loading with methotrexate. The surface properties of these films were evaluated by contact angle and spreading rate measurements. The sessile drop methods along with the thermodynamic parameter of work of adhesion were determined using the Young–Dupré semi-empirical model. The in vitro methotrexate release was assessed at a pH of 4.5 and 7.4 simulating physiological conditions. Data from the resulting profiles were fitted to the order one, Higuchi, Peppas–Sahlin and Korsmeyer–Peppas kinetic models. The results suggest a strong relationship between the thiolation degree and hydrophilic surface properties such as contact angle and water spreading rate, whereas the work of adhesion was not significantly affected. Further, these polymer films could control the methotrexate release through diverse mechanisms such as diffusion and relaxation depending on the thiolation degree and the aqueous medium employed. In fact, as thiolation degree increased, the release mechanism shifted from a primary diffusional type towards a predominant relaxation-driven mechanism. These polymer films could be used as modified systems for anticancer local delivery.

## 1. Introduction

Nowadays, cancer is a major public health problem with more than 10 million of new diagnosed cases each year. It is estimated that by 2030 it will be responsible for ~13.1 million deaths [1]. Methotrexate (MTX) is a widely used antineoplastic drug employed for the treatment of several anticancer types [2,3]. However, the oral administration of such drug has shown several drawbacks, such as high variability in bioavailability and causes a high gastrointestinal and hepatic toxicity. Likewise, the intravenous administration of MTX intensifies side effects and the latent systemic toxicity [4]. However, different strategies have been created to treat this disease reducing the risk of systemic toxicity. These imply several polymer-based drug delivery systems, which could increase the bioavailability of chemotherapeutic agents in cancer tissues, increasing their solubility and reducing the systemic side effects [5,6]. Thus, diverse delivery devices such as microspheres, patches, polymer films, osmotic pumps, liposomes, polymer-drug conjugates [7] have been developed to reach high drug levels on target tissues [8,9]. Particularly, polymer films have been characterized for controlling drug release by intratumoral implantation or release nearby a cancerous tissue. Therefore, polymer films can be used as topical or transdermal administration systems, reaching adequate drug levels within the target tissue and thus avoiding most of the side effects associated with these types of anti-cancer drugs [10]. These delivery systems can be developed from natural or synthetic polymers such as polyanhydrides, polycarbonates, polyesters, caprolactones, sodium alginates, hyaluronic acid, dextran and chitosan [8]. In this scenario, chitosan (CH) has become a promising and desirable biodegradable polymer useful for the development of anticancer medicines due to its mucoadhesivity and gelling properties allowing for a controlled release of drugs [11]. Furthermore, the chemical modifications of the CH backbone render a material with a broad range of new characteristics [12]. For instance, the addition of thiol groups allows for the generation of thiolated materials with enhanced gelling and mucoadhesive properties as compared to the parent CH [13]. This feature could modulate the release of anticancer drugs and be useful as a novel material for drug delivery from polymer films. To date, there are no reports on the use of these polymer films for the local delivery of methotrexate. Therefore, the goal of this study was to develop and characterize films of glutathione-chitosan conjugates and assess the thiolation degree on the film surface properties and release behavior employing methotrexate as anticancer model drug.

## 2. Experimental Part

### 2.1. Materials

Methotrexate (lot LRAB9958) was purchased from Sigma Aldrich (Saint Louis, MO, USA). Potassium dihydrogen phosphate (lot AMO453073342), potassium hydrogen phosphate (lot AO183904125), acetic acid (lot K41575763), sodium hydroxide (lot B1315798639) and potassium hydroxide (lot BO484233) were obtained from Merck (Darmstadt, Germany). Glutathione-chitosan conjugates with thiolation degrees of 4.4%, 5.1% and 7.0% were previously synthesized, characterized and named as CH-SH-4.4%, CH-SH-5.1% and CH-SH-7.0%, respectively [14]. A type I water was used from a purification system (Arium pro Sartorius Stedim biotechnology VF, Göttingen, Germany) for contact angle measurements.

### 2.2. Production of Polymer Films

**Figure 1:**
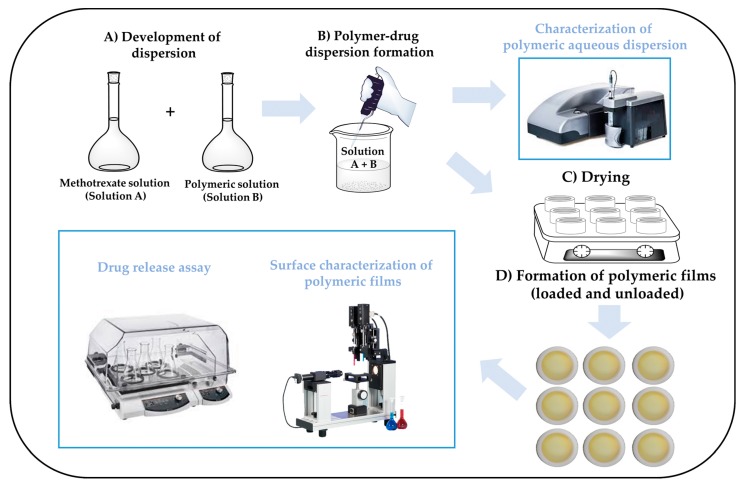
Schematics depicting the production and characterization of films of glutathione-chitosan conjugates. (**A**) Development of polymer dispersions, (**B**) formation of polymer-drug dispersion, (**C**) drying of polymer-drug dispersions, (**D**) formation of polymer films and characterization.

The development of polymer films was carried out in several steps, as shown in Figure 1. First, a 5 mg/mL solution of methotrexate in 0.1 N NaOH was prepared. Subsequently, an aliquot of 0.5 mL of this solution was added to 5.5 mL of each dispersion glutathione-chitosan conjugate at a concentration of 3 mg/mL in 1% (*v*/*v*) acetic acid under constant magnetic stirring. The resulting mixture was poured onto cylindrical Teflon molds (height = 1 cm, inside diameter = 3.5 cm and outside diameter = 4.5 cm) and dried at 40 °C for 72 h. As control, a mixture of 0.5 mL of methotrexate solution and 5.5 mL of 1% (*v*/*v*) acetic acid was poured onto the Teflon molds and prepared in the same way as polymer films.

### 2.3. Characterization of Polymer Dispersions

The particle size, polydispersity index and zeta potential of thiolated chitosan conjugates aqueous dispersions were assessed on a Zetasizer (Nano ZSP, Malvern Instrument, Worcestershire, UK). A 2 mg/mL polymer dispersion was tested at a pH value of 4. Measurements were performed in triplicate at 25 °C.

### 2.4. Surface Characterization of Polymer Films

#### 2.4.1. Contact Angle 

This is a widely used parameter to describe the spreading phenomenon of a liquid (in this case, ultrapure water) on a solid surface [15]. For instance, values of θ_c_ < 90° and 90° to 150° indicate hydrophilic and hydrophobic characteristics, respectively [16,17]. Therefore, these three polymers have a hydrophilic character due to the low magnitude of θ_c_ values as described previously [18,19,20]. Briefly, 3 mg/mL of glutathione-chitosan conjugate were dispersed in 1% (*v*/*v*) acetic acid and poured on a glass slide and oven-dried at 40 °C. The dry films thus formed on the slide surface were tested for the static contact angle. The sessile drop method was conducted a contact angle meter (OCA15EC Dataphysics Instruments, Filderstadt, Germany) coupled with a software driver (Vs. 4.5.14 SCA20, DataPhysic Instruments GmbH, Filderstadt, Germany). Data capture was recorded on an imaging development system IDS video camera, where the image information ranging from 400 to 800 frames was taken as a reference point for the static angle. Moreover, the point capture of contact angle was defined as the reflected light of the incident drop when completely disappeared (1 s, after ejection). Drop volumes ranged from 5 × 10^−3^ to 15 × 10^−3^ mL, and the height of the pouring liquid was fixed to 1 cm for all assays. All measurements were carried out at 22 ± 1 °C and 60% ± 5% relative humidity. Each measured was conducted in triplicate on different sites of the film surface. 

#### 2.4.2. Water Spreading Rate 

Variation of contact angle as a function of the elapsed time (drop age) on the film surface is an indirect measurement of the water sorption rate on the film surface. These measurements were carried out by the dynamic contact angle function provided by the Dataphysics^®^ software (OCA15EC, SCA21 software, DataPhysic Instruments GmbH, Filderstadt, Germany). Thus, the average contact angle of three measurements per second (*θ_c_*) as a function of time (s) were collected using the GraphPad Prism^®^ 8 software. 

#### 2.4.3. Thermodynamic Work of Adhesion (W_adh_)

This measurement was determined for each glutathione-chitosan conjugate film using the Young–Dupré model [21] and ultrapure water was used as the reference liquid. According to that, work of adhesion (*W_adh_*) is defined as:

(1) $$W_{adh}=\gamma_{LV}\left(Cos\;\theta_{c}+1\right)$$

where, *γ_LV_* corresponds to a test liquid surface tension in mN/m, and *θ_c_* is the contact angle between the liquid and the film surface. The Young–Dupre equation allows one to obtain information about energy issues in the in the solid-liquid interface area, establishing a relationship between the contact angle *θ_c_* and the water spreading phenomenon.

### 2.5. In Vitro Release of Methotrexate

Molds containing dry polymer films were poured in a 100 mL beaker containing 80.0 mL of phosphate buffer (pH of 4.5 or 7.4, having and ionic strength of 0.15 M adjusted with potassium chloride). The release studies were conducted on a shaker (Unimax 1010, Heidolph Instruments, Schwalbach, Germany) operated at 37 °C and 50 rpm. Subsequently, 0.3 mL aliquots were periodically taken for MTX determination and replaced by the same volume of fresh medium. Aliquots were diluted to 1.0 mL with medium and the amount of MTX released was determined by UV spectrophotometry (UV-1800, Shimadzu, Milton Keynes, UK) at 305 nm by interpolation using a calibration curve built at concentrations of 1.0, 2.0, 4.0, 6.0, 8.0, 10.0, 15.0, 20.0, 25.0 y 30.0 µg/mL. Release profile data were reported as the area under the curve (AUC) [22] and mean residence time (MRT) within the films [23]. In addition, the difference or similarity of methotrexate release profiles from the polymeric films was assessed by the calculation of the *f*_2_ similarity factor [24]. Further, the drug release from polymeric matrices was derived from different mechanisms such as diffusion, polymeric relaxation or erosion. In this context, the release mechanism of MTX from films was evaluated by fitting the release data to several semi-empirical kinetic models as follows:

*Order one model*: This model describes the release of water-soluble drugs from porous matrixes in a proportional way to the amount of drug remaining in its interior, which the drug release rate diminishes in the time. This model is expressed by the equation:

(2) $$LogQ_{t}=LogQ_{0}-\;\frac{k_{1}}{2.303}t$$

where *Q_t_* is the amount of dissolved drug at time t, *Q_o_* is the initial amount of drug in the solution and *k*_1_ corresponds to the constant of first order release [25,26].

*Higuchi model*: This model is used to describe the release of soluble and sparingly soluble drugs in aqueous medium, from diverse semi-solid and/or solid matrixes. The drug release is mediated by a diffusion process. This model is represented by the equation:

(3) $$Q_{t}=k_{H}t^{1/2}$$

where *k_H_* is the Higuchi dissolution constant, while *Q_t_* corresponds to the amount of dissolved drug at time t [27].

*Korsmeyer–Peppas model*: This model is widely used to explain the drug release mechanism from polymeric matrixes. In this way, it considers that the diffusion and erosion and/or dissolution of the matrix processes can take place. This model is expressed by the equation:

(4) $$\frac{M_{t}}{M_{\infty }}=k_{r}t^{n}$$

where $M_{t}/M_{\infty }$ corresponds to the fraction of drug released at time t, *k_r_* is the release constant, while *n* is the exponent that depends on the release mechanism and the shape of the matrix tested. For instance, in films, n values equaling 0.5 indicate a drug release driven by Fickian diffusion (Case I). Moreover, values of n between 0.5 and 1.0 suggest an anomalous transport (non-Fickian). Values higher than 1.0 mean a release controlled by the relaxation of polymeric chains (Super case II transport) [28,29].

*Peppas–Sahlin model*: This model describes the amount of diffusional or relaxational contributions from polymeric matrixes in the drug release. On this form, the model is expressed according the equation:

(5) $$\frac{M_{t}}{M_{\infty }}=k_{d}t^{0.5}+k_{r}t^{1.0}$$

where the first term of the equation is related to the Fickian (diffusional), the second term is associated to the relaxational (or erosional) contribution to drug release. *k_d_* and *k_r_* are the rate constants of diffusional and relaxational process, respectively [30].

### 2.6. Statistical Analysis

Statgraphics software v. 18 (StatPoint, Inc, The Plains, WV, USA) was used to fit the release data through the least square method. Minitab^®^ v. 18 software (Minitab^®^ Inc., State College, PA, USA) was employed for statistical comparisons using the ANOVA test, where the effect of thiolation degree on contact angle, thermodynamic work of adhesion and extent of MTX release was evaluated. The Tukey post hoc test was utilized to determine significant differences between the independent variables. 

## 3. Results and Discussion

### 3.1. Characterization of Polymer Dispersions

Results of size, polydispersity and zeta potential analyses for each glutathione-chitosan conjugate with different thiolation degrees in aqueous dispersion are shown in Table 1.

The CH-SH-5.1% sample showed the largest size and polydispersity index in aqueous media, indicating a high tendency for the chitosan polymeric particle aggregation forming a wide particle population size. Conversely, the CH-SH-4.4% and CH-SH-7.0% samples tend to form more compact aggregates and slightly less polydispersed populations. On the other hand, the zeta potential of CH-SH-5.1% was low, whereas CH-SH-4.4% and CH-SH-7.0% materials showed higher and comparable values. These results indicate that the polymer system with the lowest zeta potential (CH-SH-5.1%) has a greater tendency for inter-polymer aggregation [31]. Therefore, such polymer tends to increase the particle size in a random manner, due to lack of long-range electrostatic repulsions. On the contrary, systems with higher zeta potentials show a slightly more compact and less poly-dispersed structure. All these results suggest a non-linear relationship between the thiolation degree, and the physicochemical properties of the polymer dispersed in aqueous systems. Therefore, the thiolation degree is responsible of having different interaction degrees between the conjugated chitosan chains.

### 3.2. Surface Characterization of Glutathione-Chitosan Conjugates

#### 3.2.1. Contact Angle and Work of Adhesion Measurements

**Figure 2:**
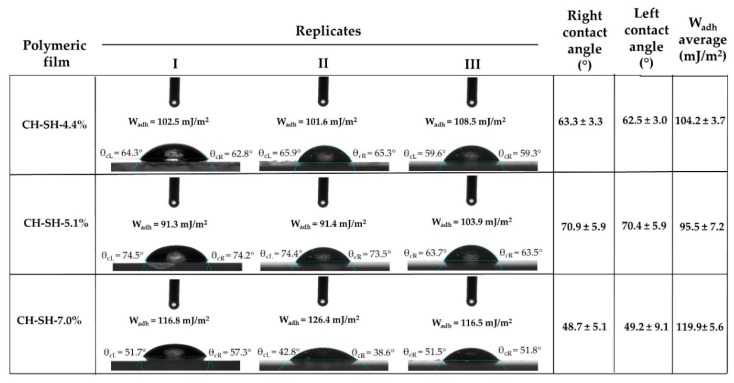
Variation of the contact angle and thermodynamic work of adhesion (mJ/m^2^) between ultra-pure water and films of glutathione-chitosan conjugates with different thiolation degrees.

Results of contact angle and the work of adhesion generated between ultra-pure water and the surface of glutathione-chitosan conjugate films are shown in Figure 2.

The three polymer films described a hydrophilic character regardless of the polymer thiolation degree. Further, each sessile drop was characterized for having symmetrical contact angles on both sides. This was reflected in the proximity of the values obtained in both drop sides indicating a very uniform film surface, allowing for a homogenous water spreading on these films. Although, there was not a linear relationship between the thiolation degree with respect to the contact angle and work of adhesion, CH-SH-5.1% showed a significantly distinct contact angle and work of adhesion as compared to other materials, indicating change in the polymer structure. This result agrees with those previously shown by the physicochemical characterization in aqueous medium, indicating a divergent spatial configuration resulted from the chitosan backbone and the glutathione side chain interactions [32,33].

#### 3.2.2. Water Spreading Rate 

**Figure 3:**
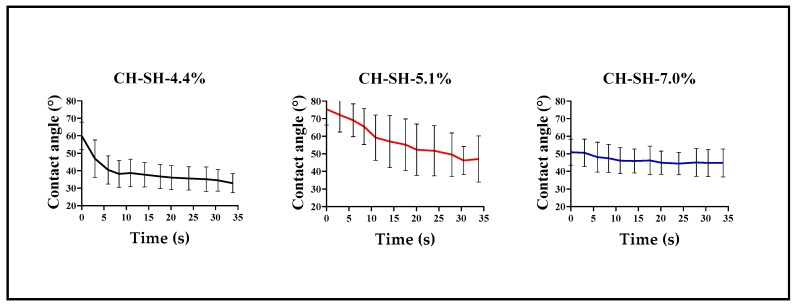
Variation of contact angle as a function of time for glutathione-chitosan conjugate films having different thiolation degrees.

The water absorption rate by the surfaces of polymeric films are depicted in Figure 3. 

The results showed that the maximal spreading change was given by the CH-SH-5.1% system, whereas CH-SH-4.4% and CH-SH-7.0% described the lowest spreading rates. These findings are unexpected, since it was expected a greater water spreading with higher thiolation degrees. However, these results are consistent with the previous zeta potential, particle size and polydispersity outcomes (Table 1), where CH-SH-5.1% exhibited the largest values indicating a high polydispersity with a high hydrophobic aggregation tendency. For this reason, it is feasible that water spreading started in a hydrophilic contact angle zone and this sample presented the largest variability. In addition, these phenomena are consistent with previous published results [14], where CH-SH-4.4% and CH-SH-7.0% materials had a net structural conformation via disulfide bonds. These bonds are formed between disulfide groups of contiguous and adjacent polymer chains [13], conferring a network rearrangement which limits water penetration into the polymer films. Likewise, CH-SH-5.1% did not show strong interactions via disulfide bonds, which was responsible for the high absorption rate. Additionally, CH-SH-5.1% has a porous structure with a high presence of mesopores (Supplementary material) that favor the water absorption. Although, no statistically significant differences were found between these values, this result suggests that these polymer films are possibly formed by random networks having multiple spatial configurations given by the diverse moieties present in the structure of the polymer material [32,33]. 

**Figure 4:**
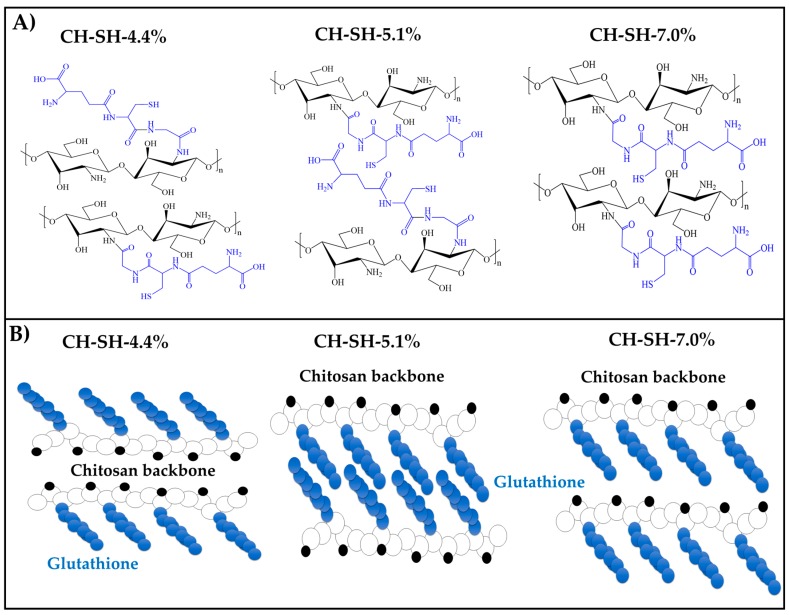
(**A**) Schematic of the possible 3D conformation of glutathione-chitosan conjugates. (**A**) Chemical structure (glutathione highlighted in blue). (**B**) Plausible configurations of the modified polymer.

Figure 4 depicts the possible spatial configuration of the polymers and summarizes all the previous determinations considering the results of thermodynamic surface properties, water spreading rate and polymer behavior in aqueous media. It is worthy to mention that CH-SH-4.4% seems to form the most hydrophilic film having the fastest water spreading rate, due to the glutathione substituent distribution in the outer chitosan backbone. Conversely, the CH-SH-5.1% was the most hydrophobic material having a slower spreading rate due to the distribution of glutathione substituents within the chitosan backbone. Further, CH-SH-7.0% was the material which showed a stationary or equilibrium state for the water spreading rate, suggesting that the spatial distribution of the glutathione substituents is randomly present inside and outside chitosan backbone. 

These results indicate that thiolation played a major role in the surface properties of the films and aqueous properties of conjugated materials. These characteristics could affect the drug release profiles as will be discussed in the following section.

### 3.3. Evaluation of the In Vitro Release of Methotrexate

**Figure 5:**
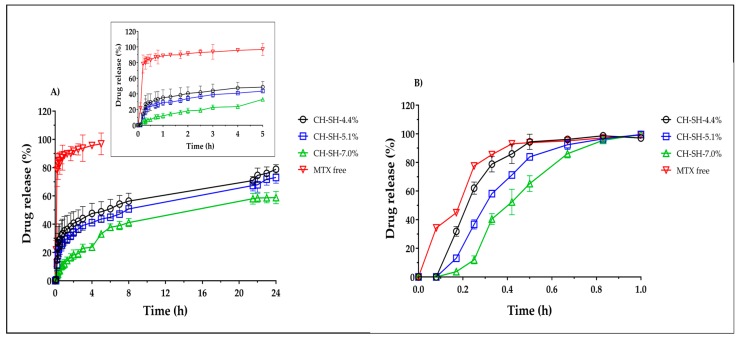
Methotrexate release profiles from films of glutathione-chitosan conjugates at 37 °C and 50 rpm. (**A**) pH of 4.5; (**B**) pH of 7.4.

The release profiles of sodium MTX from films of glutathione-chitosan conjugates are illustrated in Figure 5. It is evident the reduction of MTX release once incorporated into the films independent of the media pH. Nonetheless, the extent of MTX release at pH of 7.4 was greater as compared to that of pH of 4.5. This phenomenon is explained by the acidic nature of MTX (pKa of 4.7–5.5) [34], which remains ionized at pH of 7.4. Interestingly, at acidic MTX released showed a lag time, especially in the first 30 min since during this period drug release was lower than 40% indicating a modified release [35]. Once surpassed this time, drug release reached 50% to 80% within 24 h.

On the other hand, Table 2 shows the area under curve (AUC) and mean residence time (MRT) values of free MTX and that incorporated into glutathione-chitosan conjugate films. The AUC values from the films are much lower as compared to that of free MTX, proving that glutathione-chitosan conjugates modulate the drug release independent of the media pH. This finding was reflected on the MRT values, which were higher for polymer films than those of free MTX, indicating a reduction of the drug release rates. Likewise, MTX release was highly dependent on the thiolation degree of chitosan and thus, an inversely relationship was found between the thiolation degree and the AUC. These results agreed with those obtained for water absorption rates, where CH-SH-4.4% showed the highest absorption and thus a high MTX release. Conversely, CH-SH-7.0% showed the lowest absorption and thus a low MTX release and CH-SH-5.1% due to its rearrangement rendering hydrophobic domains, resulting in an intermediate release compared to CH-SH-4.4% and CH-SH-5.1%.

Moreover, the *f*_2_ values of the polymer films are summarized in Table 3. Values between 50 and 100 indicate similarity between the release curves, whereas lower values are considered significantly different. Results indicate that the drug release profiles of the three glutathione-chitosan conjugate film are different at pH of 7.4. Likewise, the highest thiolation degree rendered the most differentiated value among the films (*f*_2_ value near to 0). On the other hand, CH-SH-4.4% and CH-SH-5.1% showed similar release profiles at acidic pH, whereas CH-SH-7.0% exhibited a distinct profile. These findings agree with the AUC and MRT values since the increase of the thiolation degree caused a modulation of MTX release.

In order to elucidate the possible drug release mechanism from the polymer films, a kinetic analysis was performed using diverse heuristic models. The resulting parameters from the models that showed the best data fit are summarized in Table 4. 

The release rate constant (k) depended on the thiolation degree and pH employed. Interestingly, release rates were higher at a pH of 7.4 independent of the model employed. Particularly, free MTX at a pH of 7.4 showed a good fit to the order 1, indicating a release dependence on drug load. The same behavior was found for CH-SH-4.4% and CH-SH-5.1% at the same pH. Conversely, at acidic pH, these two films showed good fit to the modified Korsmeyer–Peppas model suggesting a lag time of less than 6 min driven by the decreased of drug solubility, which is explained by the prevalence of the non-ionized form of the drug at this pH. Further, CH-SH-7.0% also showed a lag time at this pH, but also showed a good fit to the Higuchi model indicating a dependence on the square root of time. In fact, at acidic pH, n values were lower than 0.5 signifying an MTX release mainly driven by the chemical potential gradient. In this scenario, the relaxation time of the polymer film was much shorter than the diffusion time for water transport [36]. Likewise, at a pH of 7.4 data showed a good fit to the Korsmeyer–Peppas model and n values ranged from 0.5 and 1 indicating an anomalous release mechanism where there is a relaxational/gelling component along with a diffusional mechanism enhanced by the drug solubility at this pH.

**Figure 6:**
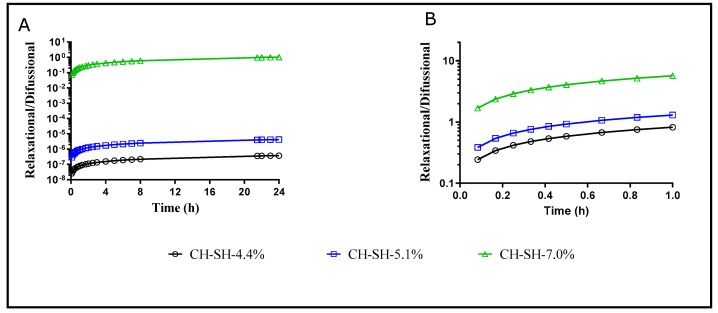
relaxational to diffusional ratio as a function of time for methotrexate release. (**A**) pH of 4.5; (**B**) pH of 7.4.

Despite the Peppas–Sahlin model’s lower degree of fitness to the data, it was utilized for understanding the magnitude and contribution of the relaxational and diffusive mechanisms responsible for drug release. Interestingly, at acidic pH, the relaxational or gelling behavior of the films was virtually absent due to the high solubility and erosion of the glutathione-chitosan conjugates resulting in a free path for drug diffusion into the media [37]. On the contrary, at a pH of 7.4 the relaxational and gelling contribution of the polymer was the predominant mechanism for drug release despite of the expected higher solubility of MTX into this media [38]. The ratio of the relaxational to the diffusional behavior at a pH of 7.4 is depicted in Figure 6. As expected at acidic pH the diffusional mechanism prevailed being larger for films having high thiolation degrees. Further, the R/F ratio increased with time, indicating that relaxation of polymer chains was the complementary process to diffusion, especially at nearly neutral pH. Likewise, at acidic, the R/F ratio is nearly constant after 4h, meaning that the maximum polymer relaxation/gelling was reached. Conversely, at a pH of 7.4 there was a possessive relaxational contribution over time. The results suggesting that the films must be employed preferably in cancer tissue or directly in skin tumor where the surrounding ambient pH is acidic [39,40].

## 4. Conclusions

One of the most important aspects to highlight in the preparation of the films corresponds to the low solubility of modified chitosan polymers, both in alkaline aqueous medium and in organic solvents. Such a condition is, in fact, a serious experimental inconvenience, because the solvent options for the preparation of these systems are very limited. In addition, such polymers describe a thermodynamic tendency to aggregation, which can lead to the formation of different conformations in the films, which also becomes another serious inconvenience in the elaboration of such films. Therefore, it is necessary to project more studies focused on collecting information on the effect of the polymer substitution degree on film formation, as well as the effect of the process conditions used during such elaboration. On the other hand, the glutathione-chitosan conjugates generated polymer films with hydrophilic surfaces, where the surface of the CH-SH-4.0% system proved to be the most hydrophilic, CH-SH-5.1% the more hydrophobic, and CH-SH-7.0% showed a dual hydrophobic character. On the other hand, a marked trend was not found between the thermodynamic parameters, contact angle and W*_adh_* with respect to the thiolation degree, indicating that the polymer films are formed by different ways of interpolymer interactions, suggesting a different spatial distribution for glutathione substituents in the chitosan backbone. The release mechanism depended on the thiolation degree and pH employed, where the release rate decreased as the thiolation degree increased. A Diffusional mechanism was mainly responsible for drug release at acidic pH, whereas a relaxational/gelling component was also present at nearly neutral pH. The relaxational component increased with thiolation degree modulating MTX release.

## Figures and Tables

**Table 1:** Physical properties of glutathione-chitosan in aqueous dispersion.

Polymer Material	Size (nm)	Polydispersity Index	Zeta Potential (mV)
CH-SH-4.4%	1102.5 ± 20.1	0.70 ± 0.08	+23.0 ± 1.2
CH-SH-5.1%	3424.7 ± 38.4	0.91 ± 0.08	+15.1 ± 0.7
CH-SH-7.0%	551.2 ± 17.3	0.76 ± 0.07	+21.1 ± 1.7

**Table 2:** Area under curve (AUC) and mean residence time (MRT) values of MTX released from polymer films at different pH values.

Polymer	AUC		MRT	
	pH = 4.5	pH = 7.4	pH = 4.5	pH = 7.4
MTX free	464.0	80.0	1.03	0.17
CH-SH-4.4%	198.8	73.7	1.33	0.16
CH-SH-5.1%	170.1	65.2	1.34	0.17
CH-SH-7.0%	94.2	55.7	1.34	0.18

Values calculate at 1h and 5h for pH 4.5 and 7.4, respectively.

**Table 3:** Similarity factor (*f*_2_) of polymer films at different pH values.

Release Profiles	*f*_2_ Factor	
	pH = 4.5	pH = 7.4
CH-SH-4.4%-CH-SH-5.1%	60.0	43.8
CH-SH-5.1%- CH-SH-7.0%	34.5	43.8
CH-SH-4.4%-CH-SH-7.0%	29.3	29.1

**Table 4:** Fitting parameters of several kinetic models of MTX release.

Polymer	pH	Order 1		Higuchi		Korsmeyer–Peppas with t-lag				Peppas–Sahlin		
		k_1_	r^2^	k_H_	r^2^	k	n	t-lag	r^2^	k_d_	k_r_	r^2^
MTX free	4.5	0.500	0.6835	29.226	0.6830	-	-	-	-	-	-	-
	7.4	4.497	0.9546	105.1	0.9353	-	-	-	-	-	-	-
CH-SH-4.4%	4.5	0.050	0.9070	13.088	0.8937	0.342	0.23	0.10	0.9800	0.19	1.5 × 10^−8^	0.8936
	7.4	4.822	0.9683	121.8	0.8576	1.24	0.64	0.08	0.9654	1.05	0.86	0.8327
CH-SH-5.1%	4.5	0.044	0.9220	12.876	0.9322	0.286	0.27	0.10	0.9819	0.17	1.5 × 10^−8^	0.9321
	7.4	5.178	0.9470	125.1	0.8950	1.24	0.64	0.08	0.9305	0.496	0.644	0.8822
CH-SH-7.0%	4.5	0.036	0.9385	12.888	0.9807	0.23	0.24	0.0002	0.9731	0.07	0.015	0.9356
	7.4	5.094	0.8667	123.6	0.8622	1.25	0.88	0.08	0.8892	0.16	0.910	0.9115

n: correspond to the release exponent, - indicates no fitting to these models.

## Supplementary Materials

The following are available online at https://www.mdpi.com/2073-4360/11/12/2032/s1.

## References

[1] Senapati S., Mahanta A.K., Kumar S., Maiti P. (2018). Controlled drug delivery vehicles for cancer treatment and their performance. Signal Transduct. Target. Ther..

[2] Jolivet J., Cowan K.H., Curt G.A., Clendeninn N.J., Chabner B.A. (1983). The Pharmacology and Clinical Use of Methotrexate. N. Engl. J. Med..

[3] Galluzzi L., Vacchelli E., Bravo-San Pedro J.M., Buqué A., Senovilla L., Baracco E.E., Bloy N., Castoldi F., Abastado J.P., Agostinis P. (2014). Classification of current anticancer immunotherapies. Oncotarget.

[4] Chandak A.R., Verma P. (2008). Design and development of hydroxypropyl methylcellulose (HPMC) based polymeric films of methotrexate: Physicochemical and pharmacokinetic evaluations. Pharm. Soc. Jpn..

[5] Sonvico F., Barbieri S., Colombo P., Mucchino C., Barocelli E., Cantoni A.M., Cavazzoni A., Petronini P.G., Rusca M., Carbognani P. (2018). Physicochemical and pharmacokinetic properties of polymeric films loaded with cisplatin for the treatment of malignant pleural mesothelioma. J. Thorac. Dis..

[6] Ahmed T.A., Aljaeid B.M. (2016). Preparation, characterization, and potential application of chitosan, chitosan derivatives, and chitosan metal nanoparticles in pharmaceutical drug delivery. Drug Des. Devel. Ther..

[7] Moses M.A., Brem H., Langer R. (2003). Advancing the field of drug delivery. Cancer Cell.

[8] Wolinsky J.B., Colson Y.L., Grinstaff M.W. (2012). Local drug delivery strategies for cancer treatment: Gels, nanoparticles, polymeric films, rods, and wafers. J. Control. Release.

[9] Sgorla D., Almeida A., Azevedo C., Bunhak E.J., Sarmento B., Cavalcanti O.A. (2016). Development and characterization of crosslinked hyaluronic acid polymeric films for use in coating processes. Int. J. Pharm..

[10] Marchetti J.M., de Souza M.C., Marotta-Oliveira S.S. (2011). Nanocarriers and cancer therapy: Approaches to topical and transdermal delivery. Nanocosmetics and nanomedicines.

[11] Wimardhani Y.S., Suniarti D.F., Freisleben H.J., Wanandi S.I., Siregar N.C., Ikeda M.A. (2014). Chitosan exerts anticancer activity through induction of apoptosis and cell cycle arrest in oral cancer cells. J. Oral Sci..

[12] Patil O., Schulz D.N. (1998). Functional Polymers: An overview. ACS Symposium Series.

[13] Rojas J., Ciro Y., Salamanca C. (2017). Thiolated Chitosan: A Promising Strategy for Improving the Effectiveness of Anticancer Drugs. Analytical and Pharmaceutical Chemistry.

[14] Ciro Y., Rojas J., Yarce C.J., Salamanca C.H. (2019). Preparation, characterization and rheological behavior of glutathione-chitosan conjugates in aqueous media. Appl. Rheol..

[15] Rojas Y., Ciro Y., Constain S. (2017). Effect of the degree of acetylation on the physical and tableting properties of chitin. Chitin: Properties, Applications and Research.

[16] Zhang D., Flory J.H., Panmai S., Batra U., Kaufman M.J. (2002). Wettability of pharmaceutical solids: Its measurement and influence on wet granulation. Colloids Surf. A Physicochem. Eng. Asp..

[17] Hejazi I., Sadeghi G.M.M., Jafari S.H., Khonakdar H.A., Seyfi J., Holzschuh M., Simon F. (2015). Transforming an intrinsically hydrophilic polymer to a robust self-cleaning superhydrophobic coating via carbon nanotube surface embedding. Mater. Des..

[18] De Gennes P.G. (1985). Wetting: Statics and dynamics. Rev. Mod. Phys..

[19] Chau T.T., Bruckard W.J., Koh P.T.L., Nguyen A.V. (2009). A review of factors that affect contact angle and implications for flotation practice. Adv. Colloid Interface Sci..

[20] Li S., Zhai S., Liu Y., Zhou H., Wu J., Jiao Q., Zhang B., Zhu H., Yan B. (2015). Experimental modulation and computational model of nano-hydrophobicity. Biomaterials.

[21] Kwok D.Y., Neumann A.W. (1998). Contact angle measurement and contact angle interpretation. Adv. Colloid Interface Sci..

[22] Anderson N.H., Bauer M., Boussac N., Khan-Malek R., Munden P., Sardaro M. (1998). An evaluation of fit factors and dissolution efficiency for the comparison of in vitro dissolution profiles. J. Pharm. Biomed. Anal..

[23] Podczeck F. (1993). Comparison of in vitro dissolution profiles by calculating mean dissolution time (MDT) or mean residence time (MRT). Int. J. Pharm..

[24] Diaz D.A., Colgan S.T., Langer C.S., Bandi N.T., Likar M.D., Alstine L.V. (2016). Dissolution similarity requirements: How similar or dissimilar are the global regulatory expectations?. AAPS J..

[25] Siepmann J., Siepmann F. (2013). Mathematical modeling of drug dissolution. Int J Pharm.

[26] Marcos B., Bruschi M.L. (2015). Mathematical models of drug release. Strategies to Modify the Drug Release from Pharmaceutical Systems.

[27] Higuchi W.I. (1967). Diffusional models useful in biopharmaceutics drug releaserate processes. J. Pharm. Sci..

[28] Ritger P.L., Peppas N.A. (1987). A simple equation for description of solute release II. Fickian and anomalous release from swellable devices. J. Control. Release.

[29] Ritger P.L., Peppas N.A. (1987). A simple Fickian equation for description of solute release. I. Fickian and non-Fickian release from non-swellable devices in the form of slabs, spheres, cylinders or discs. J. Control. Release.

[30] Peppas N.A., Sahlin J.J. (1989). A simple equation for the description of solute release. III. Coupling of diffusion and relaxation. Int. J. Pharm..

[31] Bhattacharjee S. (2016). DLS and zeta potential—What they are and what they are not?. J. Control. Release.

[32] Ramya R., Sudha P.N., Mahalakshmi J. (2012). Preparation and characterization of chitosan binary blend. Int. J. Sci. Res. Publ..

[33] Kafedjiiski K., Föger F., Werle M., Berknkop-Schnürch A. (2005). Synthesis and in vitro evaluation of a novel chitosan-glutathione conjugate. Pharm. Res..

[34] Jang J.H., Jeong S.H., Lee Y.B. (2019). Preparation and in vitro/in vivo characterization of polymeric nanoparticles containing methotrexate to improve lymphatic delivery. Int. J. Mol. Sci..

[35] Costa P., Sousa L. (2001). Modeling and comparison of dissolution profiles. Eur. J. Pharm. Sci..

[36] Rojas J., Ciro Y. (2016). Evaluation of the swelling and diffusional behavior of guar gum for the controlled release of bioactive agents. Advances in Medicine and Biology.

[37] Hancock B.C., Zografi G. (1994). The relationship between the glass transition temperature and the water content of amorphous pharmaceutical solids. Pharm. Res..

[38] Sreenivs S.A., Pai K.V. (2008). Thiolated chitosans: Novel polymers for mucoadhesive drug delivery—A review. Trop. J. Pharm. Res..

[39] Liu J., Huang Y., Kumar A., Tan A., Jin S., Mozhi A., Liang X.J. (2014). pH-Sensitive nano-systems for drug delivery in cancer therapy. Biotechnol. Adv..

[40] Du J., Lane L.A., Nie S. (2015). Stimuli-responsive nanoparticles for targeting the tumor microenvironment. J. Control. Release.

