# Supplementary material for: Production and Characterization of Glutathione-Chitosan Conjugate Films as Systems for Localized Release of Methotrexate
Source: Polymers (Basel). 2019 Dec 7;11(12):2032. doi: 10.3390/polym11122032 (PMC6960860; doi:10.3390/polym11122032)
Supplement: Supplementary file 1 [file polymers-11-02032-s001.pdf]

Article

Production and Characterization of Glutathione-Chitosan Conjugate Films as Systems for Localized Release of Methotrexate

Yhors Ciro [1,*], John Rojas [1,] Cristian J. Yarce [2] and Constain H. Salamanca [2,*]

[1] University of Antioquia, School of Pharmaceutical and Food Sciences, Department of Pharmacy, 67 Street No. 53–108, Medellín, Colombia.); yhors.ciro@udea.edu.co (YC), jrojasca@gmail.com (JR)

[2] Laboratorio de Diseño y Formulación de Productos Químicos y Derivados, Departamento de Ciencias Farmacéuticas, Facultad de Ciencias Naturales, Universidad ICESI, Calle 18 No. 122–135, Cali, 760035, Colombia; cjyarce@icesi.edu.co (C.J.Y), chsalamanca@icesi.edu.co (C.H.S)

* Correspondence: chsalamanca@icesi.edu.co (C.H.S), yhors.ciro@udea.edu.co (YC)

Received: 25 November 2019; Accepted: 5 December 2019; Published: date

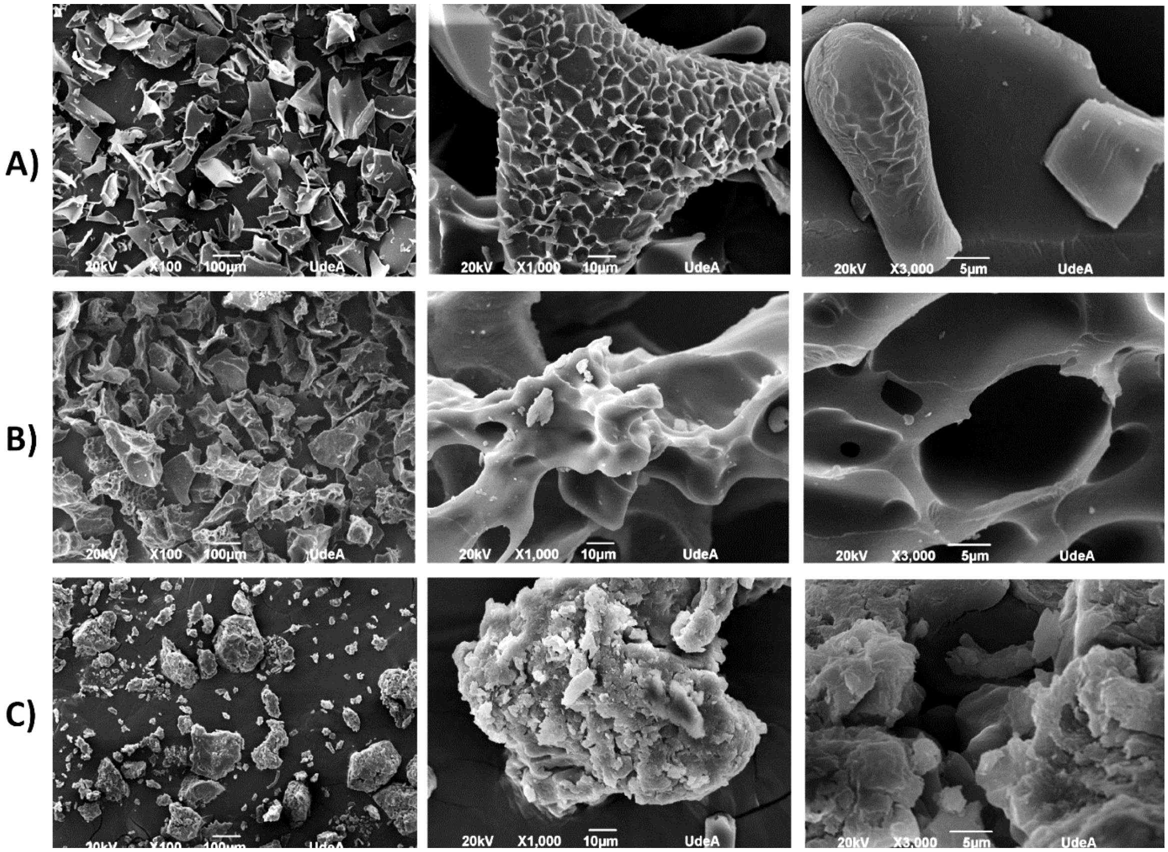

Figure S1. SEM pictures of polymers at 100x, 1000x and 3000x. A) CH-SH-4.4%, B) CH-SH-5.1% and C) CH-SH-7.0%,
